# Fibrogenesis in *LAMA2*-Related Muscular Dystrophy Is a Central Tenet of Disease Etiology

**DOI:** 10.3389/fnmol.2020.00003

**Published:** 2020-02-04

**Authors:** Anthony Accorsi, Megan L. Cramer, Mahasweta Girgenrath

**Affiliations:** ^1^Fulcrum Therapeutics, Cambridge, MA, United States; ^2^Rare Disease Research Unit, Pfizer Inc., Cambridge, MA, United States

**Keywords:** congenital muscular dystrophy, laminin, fibrosis, integrin, TGF-beta, myofibroblast, renin–angiotensin system

## Abstract

*LAMA2*-related congenital muscular dystrophy, also known as MDC1A, is caused by loss-of-function mutations in the alpha2 chain of Laminin-211. Loss of this protein interrupts the connection between the muscle cell and its extracellular environment and results in an aggressive, congenital-onset muscular dystrophy characterized by severe hypotonia, lack of independent ambulation, and early mortality driven by respiratory complications and/or failure to thrive. Of the pathomechanisms of MDC1A, the earliest and most prominent is widespread and rampant fibrosis. Here, we will discuss some of the key drivers of fibrosis including TGF-beta and renin–angiotensin system signaling and consequences of these pathways including myofibroblast transdifferentiation and matrix remodeling. We will also highlight some of the differences in fibrogenesis in congenital muscular dystrophy (CMD) with that seen in Duchenne muscular dystrophy (DMD). Finally, we will connect the key signaling pathways in the pathogenesis of MDC1A to the current status of the therapeutic approaches that have been tested in the preclinical models of MDC1A to treat fibrosis.

## Congenital Muscular Dystrophy

Congenital muscular dystrophies (CMDs) are a highly heterogeneous group of early-onset neuromuscular disorders characterized by delayed motor development, severe hypotonia, and extreme muscle wasting (Schessl et al., 2006; Collins and Bonnemann, 2010). Although the first forms of CMD were recognized as early as 1903, it took decades to link these conditions to specific gene defects. CMDs can arise from mutations in many muscle proteins but the most prevalent forms of this disease arise from deficiencies in proteins at the interface of the muscle cell membrane (sarcolemma) and its associated extracellular matrix (ECM). Like in most cells, the interaction between the ECM and the sarcolemma is critical for muscle cell survival, homeostasis, and basic organ function (Tome et al., 1994; Jimenez-Mallebrera et al., 2005; Schessl et al., 2006; Lisi and Cohn, 2007; Collins and Bonnemann, 2010; Bertini et al., 2011). It is therefore conceivable that disruption in the link between these two structures often leads to rather severe forms of muscular dystrophies.

Pathology can arise from mutated gene products within the ECM itself. For example, defects in one of the alpha chains of type VI collagen cause Ullrich CMD or Bethlem myopathy, and disruption of the alpha2 chain of Laminin-211 leads to *LAMA2*-deficient congenital muscular dystrophy (aka MDC1A). Alternatively, CMDs can arise from mutations in one of the many glycosyltransferases that add sugar moieties on alpha-Dystroglycan, an important receptor of the dystrophin–glycoprotein complex (DGC) that links the cell to the ECM. Abnormalities of *O*-mannosyl glycosylation of this key receptor result in a decrease or complete loss in the ability of alpha-dystroglycan to bind laminin, a ligand in the ECM (Tezak et al., 2003; Jimenez-Mallebrera et al., 2005; Schessl et al., 2006; Lisi and Cohn, 2007). While disruption of the DGC complex is also a consequence in Duchenne muscular dystrophy (DMD), a disease that is caused by mutations in the dystrophin gene (Lapidos et al., 2004), presentation, and clinical manifestations of CMDs differ from DMD in many respects. Patients with CMDs show signs of muscle and, in many cases, nervous system pathology at time of birth indicating a critical developmental role of the proteins involved. Additionally, children with CMD present with an early episode of severe pathology followed by a stabilization period. This is in contrast with DMD, which has a later onset but is a consistently progressive disease (Geranmayeh et al., 2010; Brogna et al., 2019). Pathological processes such as inflammation, fibrosis, and aberrant regenerative capacity appear to be conserved across all muscular dystrophies. However, they could play a much more etiological role in CMDs compared to DMD.

As previously stated, CMDs result from a large number of different genetic defects and encompass a wide range of pathophysiological processes, making it challenging to discuss them as a whole. Here we will review MDC1A, the second most prevalent form of CMD. We will particularly explore the clinical features and molecular signature of the fibrotic pathophysiology of MDC1A and discuss the therapeutic approaches that are currently being investigated to treat fibrosis in this devastating, incurable disease.

## *Lama2*-Related Muscular Dystrophy

*LAMA2*-related muscular dystrophy (MCD1A) is caused by mutations in the *LAMA2* gene, located on chromosome 6q22-q23 in humans. It encodes the alpha2 chain of laminin-211 (composed of alpha2, beta1, and gamma1 subunits) that is an essential, multi-functional ECM protein. The laminin superfamily of matrix proteins plays an integral role in multiple cellular processes such as proliferation, differentiation, migration, and cell adhesion. Laminin-211 is primarily expressed in the basal membranes of skeletal muscle and Schwann cells, as well as in capillaries between the astrocyte foot processes and vessels of the brain. Other tissues expressing laminin-211 include heart, kidney, lung, stomach, placenta, and testis. It is expressed as early as week 7 in human embryos and E11 in mice, underscoring its critical role during skeletal muscle development (Leivo et al., 1989; Quijano-Roy et al., 1993; Patton et al., 1997; Nakagawa et al., 2001; Tezak et al., 2003; Jimenez-Mallebrera et al., 2005; Holmberg and Durbeej, 2013).

Laminin-211 has a host of binding partners both in the ECM and in the cell membrane. Specifically, it interacts with the extracellular alpha subunit of dystroglycan, a transmembrane dimeric protein belonging to the DGC. Alpha-dystroglycan connects to the intracellular cytoskeleton by interacting with its beta subunit. Beta-dystroglycan binds dystophin, thus connecting the ECM to the contractile apparatus. Yet another important partner of laminin-211 is integrin-alpha7beta1, a transmembrane protein complex that links the ECM with the underlying cytoskeletal actin network, possibly by interacting with intermediate proteins such as talin and/or integrin-linked kinase. The laminin–alpha-dystroglycan interaction has been shown to activate the PI3K/Akt pathway and the binding of laminin with integrins results in activation of the focal adhesion kinase (FAK) and mitogen-activated protein kinase (MAPK) pathways. In addition to these cell surface partners, laminin-211 also has many binding partners in the ECM such as agrin, nidogen, perlecan, and collagen IV (Yurchenco and O’Rear, 1994; Straub et al., 1997; Talts et al., 1999; Lapidos et al., 2004; Tzu and Marinkovich, 2008; Gumerson and Michele, 2011).

Pathophysiology of MDC1A was first described by Tome et al. (1994) and follows a strong genotype–phenotype correlation (Geranmayeh et al., 2010). Mutations that allow for at least partial expression of the alpha2 chain of laminin display a milder phenotype when compared to severe pathology that results when there is a complete loss of this protein. Children with MDC1A show profound muscle weakness and hypotonia, either at birth or soon after. They also develop spinal deformities as well as contractures at their elbows, knees, and ankles. Serum creatine kinase levels are high in these patients but not as high as DMD patients. Although some affected children may achieve the ability to sit and stand with support, most will never ambulate independently. Brain MRIs from these patients show white matter hypodensity, though impaired brain function is not part of the MDC1A pathology. In most cases of MDC1A, affected children without palliative care die prematurely due to either respiratory complications or failure to thrive (Quijano-Roy et al., 1993; Philpot et al., 1999; Allamand and Guicheney, 2002; Tezak et al., 2003).

## Fibrosis and Inflammation

Chronic inflammation and widespread fibrosis in the interstitial space are pathological signatures of laminin-211-deficient muscles. Unlike DMD, where fibrosis develops later during the disease progression, it may play a more etiological role in driving MDC1A pathology. In children with MCD1A, there is a massive early surge of inflammation in the months soon after birth. However, after the initial inflammatory episode, fibrosis sets in and seems to be the main driver of laminin-deficient pathology rather than chronic inflammation. Limited patient data are available that show fibrosis as an early driver of MDC1A pathology; however, a study by Taniguchi et al. (2006) provided, for the first time, the data to support early dysregulation of ECM proteins in the human disease. Taniguchi et al. (2006) reported that muscle tissue from MDC1A patients exhibited extensive interstitial connective tissue and a lack of regenerating fibers as early as 20 days of age. One potential problem of very early fibrosis is its effect on myogenesis. An altered myomatrix could modify the myogenic potential of satellite cells and thus deleteriously impact postnatal muscle growth (Thomas et al., 2015). This was further supported by gene expression data in MDC1A patients which revealed overexpression of several ECM components and downregulation of muscle structural components. A similar gene expression profile was also seen in biopsies from Fukuyama muscular dystrophy, another CMD resulting from the loss of laminin–dystroglycan interaction (Taniguchi et al., 2006). Further, it has been shown that these ECM genes are not only expressed by interstitial fibroblasts but also by the muscle fibers themselves. This suggests that myofibers undergo an environment-driven transition into fibrotic effector cells, further deleteriously affecting myogeneis and driving the etiology of the disease (Pessina et al., 2015). While increased expression of ECM-related genes is also seen in DMD biopsies, they appear much later in the disease progression as a result of many failed rounds regeneration and thus are likely a secondary consequence rather than a primary disease driver as seen in CMDs. However, this needs to be confirmed with a more comprehensive study in a larger and longitudinal data set.

**FIGURE 1:**
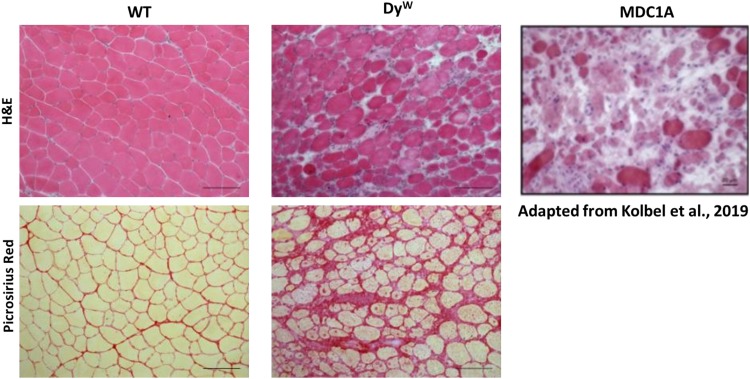
Histological analysis shows an extensive amount of fibrosis in muscles from *DyW* mice, a model for MDC1A, during early development. Tibialis anterior muscle isolated from 4 weeks old wild-type (WT) and *DyW* are stained with Hematoxylin and Eosin **(top)** and Picrosirius Red **(bottom)**. Representative images show established muscle pathology with fiber size variability, infiltrating cells, and increased interstitial space in the DyW tissue compared to the healthy WT sections **(top)**. Picrosirius red staining of the TA muscle reveals extensive deposition of collagen by 4 weeks of age in these mice. For reference, we have included an image of a muscle biopsy from a seven-year-old boy stained with Hematoxylin and Eosin (Kolbel et al., 2019).

A number of laboratories, including our own, have corroborated MDC1A patient data in various mouse models of laminin-alpha2 deficiency, further implicating fibrosis and inflammation as critical drivers of this disease. There are several mouse models for MDC1A (*dy^2*J*^*/*dy^2*J*^*, *dy/dy*, *dy^*W*^/dy^*W*^*, *dy^3*K*^/dy^3*K*^*) that display moderate to severe CMD phenotypes directly correlating with the levels of laminin-alpha2 expressed (reviewed in Gawlik and Durbeej, 2011). It is worth noting that the level of fibrosis in these models also appears to correlate with severity. Our laboratory worked primarily with the *dy^*W*^/dy^*W*^* (*DyW*) model that was generated by crossing heterozygous B6.129 *Lama2*^*dy–W/+*^ mice, which carry a targeted *DyW* mutation in the *Lama2* gene (originally generated and kindly shared by Dr. Eva Engvall, Burnham Institute, La Jolla, CA, United States). Like humans, mice that are homozygous for the *DyW* allele present with severe disease pathology characterized by accelerated muscle wasting, limited or no regenerative capacity, inflammation, and widespread fibrosis. This pathology can be observed as early as 1 day of age where there is a clear, widespread disruption of muscle architecture and increased endomysial connective tissue (Figure 1). At this time point, these muscles also show a large mononuclear cell population likely made up of some combination of unfused myoblasts, macrophages, fibroblasts, and/or other infiltrating cells, mimicking the very early rampant inflammatory response observed in children with MDC1A (Mehuron et al., 2014).

There are several pathways that could play a role in driving the fibrotic phenotype of MDC1A. We will, however, focus this review on the biology of fibrosis that is dictated by TGF-beta and renin–angiotensin system (RAS) signaling pathways since these pathways have been extensively characterized by us and others in the context of laminin-deficiency. We will also touch upon the biology of integrin-alphaV as it is intimately involved in the release of TGF-beta from its latent complex in the ECM and has been established in facilitating fibrosis and matrix remodeling in many organs.

## Tgf-Beta as a Driver of Fibrosis

The most significant driver of fibrosis in many diseases, including muscular dystrophies, is TGF-beta. This pro-fibrotic cytokine is synthesized as a precursor protein in the endoplasmic reticulum and assembled as a non-covalently bound complex of a short C-terminal disulfide-linked homodimer (the mature cytokine) and a longer N-terminal disulfide-linked homodimer that called the latency associated peptide (LAP). This small latent complex (SLC) is further non-covalently linked to another set of proteins in the endoplasmic reticulum called latent TGF-beta binding proteins (LTBPs) to form the large latent complex (LLC). The LLC is secreted from cells, and the LTBPs tether to the ECM. This latent complex confines TGF-beta to an inactive form by hiding the TGF-beta receptor binding domains, allowing for tight regulation of signaling. Activation of TGF-beta can occur through multiple routes including proteolysis, thrombospondin-1, reactive oxygen species, or pH to dissociate the LAP from the mature TGF-beta peptide (Saharinen et al., 1999; Annes et al., 2003; Shi and Massague, 2003; Leask and Abraham, 2004; Pohlers et al., 2009).

Another way in which TGF-beta can become activated/freed from the LLC is through the actions of integrins. Integrins comprise a family of 18 alpha and 8 beta proteins that come together to form 24 distinct heterodimeric, membrane-spanning proteins consisting of one alpha and one beta subunit. The alpha subunit imparts ligand specificity and the beta subunit is the effector of downstream signaling. These proteins often serve as receptors for laminins and other extra/matricellular proteins and participate in a wide array of cellular functions including migration, signal transduction, and cell stability. In particular, integrin-alphaV, along with its beta dimer partners -beta1, -beta3, -beta5, -beta6, and -beta8, has been shown to be intricately linked to TGF-beta signaling dysregulation in the progression of many diseases including cancer, heart disease, and the fibrosis of various organs (Munger and Sheppard, 2011; Worthington et al., 2011; Mamuya and Duncan, 2012; Conroy et al., 2016).

Activated TGF-beta binds to TGF-beta receptor II to form a heterotetrameric complex with TGF-beta receptor I, initiating a signaling cascade ending with activation of the transcription factor Smad2/3 and a canonical gene expression program. This program includes TGF-beta itself as well as extra/matricellular proteins including integrins, collagen isoforms, fibronectin, osteopontin, periostin, thrombospondins, and other matrix remodeling proteins including matrix metalloproteases (MMPs) (Ignotz and Massague, 1986; Leask and Abraham, 2004; Pohlers et al., 2009). This process has been shown to be upregulated in mouse models of MDC1A. Indeed, Mehuron et al. (2014) showed that phosphorylated smad2/3 was increased during the early development of *DyW* mice. This very early onset is similar to that observed in MDC1A patients. In addition to increased activity of the phosphorylated smad2/3, there was a parallel downregulation of inhibitory smad7, suggesting even further amplification of the TGF-beta signaling pathway. Downstream genes encoding extra/matricellular proteins were also upregulated including fibronectin, osteopontin, periostin, and collagen I (Mehuron et al., 2014). Increased TGF-beta signaling as well as matricellular protein expression was persistent throughout the entire postnatal development of *DyW* mice, thus demonstrating a chronically dysregulated matrix remodeling process that may be etiological to the pathology following loss of laminin-alpha2 (Accorsi et al., 2015). This is consistent with previously mentioned clinical data from MDC1A and Fukuyama muscular dystrophy patients showing significant upregulation of ECM genes indicative of active fibrosis at very early stages of disease (Taniguchi et al., 2006), supporting the hypothesis that fibrosis is at the center of CMD pathology.

## Consequences of Chronically Dysregulated Tgf-Beta Signaling: Myofibroblast Transdifferentiation

A potential consequence of chronically dysregulated TGF-beta signaling and matricellular protein upregulation is the transdifferentiation of various cell lineages into myofibroblasts. Myofibroblasts are contractile, hyper-secretory fibroblastic cells that are activated following tissue injury and are part of the normal wound healing process. Their secretions and contractile properties are critical to facilitate migration of inflammatory and tissue-specific stem cells to the site of injury as well as to induce wound closure. In the context of normal wound healing, myofibroblasts undergo mass apoptosis or de-differentiate back to their original cell types in response to decreased matrix stiffness. However, in pathological scenarios, these cells can persist and significantly exacerbate fibrotic pathology (Hinz, 2007, 2010; Hinz et al., 2007; Klingberg et al., 2013).

**FIGURE 2:**
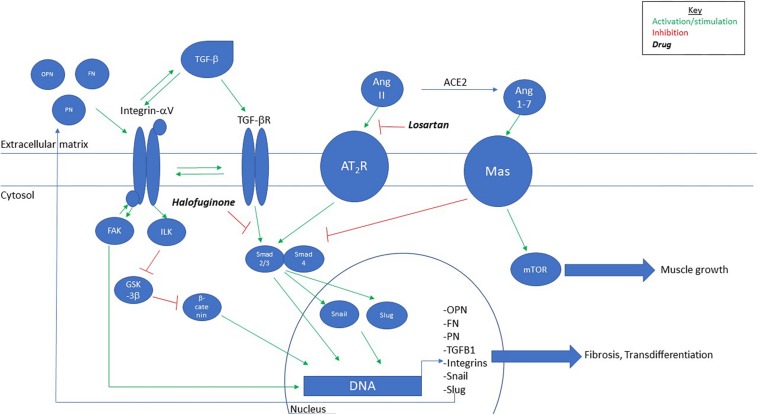
Proposed model for signaling pathway of fibrosis in MDC1A. TGF-beta, through integrin-alphaV-mediated activation, leads to upregulation of extra-/matricellular proteins that feedback to activate integrin-alphaV and further activation of TGF-beta. Chronic signaling leads to myofibroblast differentiation and fibrosis. Intervention with Losartan, Halofuginone, or Angiotensin-(1–7) inhibits TGF-beta-mediated fibrosis. OPN, osteopontin; FN, fibronectin; PN, periostin.

Multiple cell types have been shown to transdifferentiate into myofibroblasts via TGF-beta signaling via induction of epithelial-to-mesenchymal transdifferentiation (EMT)-related transcription factors (Slug, Snail, and Twist) (Figure 2). While fibroblasts are the most prominent cell type to transdifferentiate into myofibroblasts, hepatic stellate cells, smooth muscle cells, and bone marrow-derived progenitors like pericytes and fibrocytes have also been shown to undergo this transition (Mamuya and Duncan, 2012). Interestingly, myoblasts are also capable of transdifferentiating to myofibroblasts. It has been shown that C2C12 myoblasts can undergo this process in a TGF-beta, sphingosine-1 phosphate receptor3 (S1P3), Rho/Rho kinase (ROCK)-dependent pathway (Cencetti et al., 2010). Results in chronic injury models suggest that the process of myofiboblast transdifferentiation occurs in skeletal muscle and drives disease pathology in part due to dysregulation and accumulation of fibroadipogenic progenitors (FAPs). It has been shown that PDGFRalpha- and Tcf4-positive cells in denervated or mdx mice co-label with the myofibroblast marker alpha-smooth muscle actin that coincides with excessive fibrosis (Contreras et al., 2016). Additionally, it has been shown that excessive TGF-beta signaling prevents apoptosis of pro-fibrogenic FAPs during chronic injury thereby promoting a fibrotic environment (Lemos et al., 2015). These results suggest that in scenarios of chronic dysregulation of a pro-fibrotic cascade, such as in muscular dystrophies, persistent cellular identity changes away from a myogenic and toward a fibrogenic phenotype could be playing major roles in driving disease pathology.

This phenomenon has also been shown in other models of muscle fibrosis. In end-stage *mdx* mice, chronic TGF-beta signaling results in a loss of myogenic cell identity and a push toward a fibrogenic mesenchymal cell identity (Pessina et al., 2015). Likewise, we observed expression of integrin-alphaV on matured myofibers from *DyW* mice (Accorsi et al., 2015). This protein is typically only expressed in cells of a mesenchymal lineage in adult skeletal muscle (Sinanan et al., 2008; Murray et al., 2017). It is interesting to note that a similar phenomenon has been observed in aged human skeletal muscle where increased TGF-beta signaling and acetylation of the 27th residue of histone H3 (H3K27ac) on ECM genes pushes satellite cells away from myogenic fates and toward fibrogenic fates (Zhou et al., 2019). Taken together, this data demonstrate that satellite cells have the ability to become fibrotic effector cells in response to chronic TGF-beta signaling.

## Chronic Dysregulation of Tgf-Beta Signaling Leads to Defective Myogenesis

The consequences of chronically dysregulated TGF-beta signaling in muscle result in myomatrix remodeling/stiffening (Hinz, 2015) ultimately leading to impaired myogenesis and muscle function. Increased matrix stiffness has been shown to inhibit myogenesis in C2C12 myoblasts plated on stiff surfaces (Engler et al., 2004; Romanazzo et al., 2012). Additionaly, *in vivo* atomic force microscopy (AFM) measured a 50% increase in overall stiffness due to fibrosis in *mdx* mice compared to age-matched wild-type controls (Puttini et al., 2009). These studies have not been performed in models of MDC1A.

The transcription factors downstream of TGF-beta signaling that drive myofibroblast transdifferentiation have been shown to inhibit myogenic differentiation. Snail/Slug normally blocks MyoD binding to differentiation enhancer elements to prevent precocious differentiation. Overexpression of either protein, however, completely inhibits myoblast differentiation (Zhao et al., 2002; Soleimani et al., 2012). Twist has also been shown to play inhibitory roles in myogenesis. Overexpression of Twist during C2C12 differentiation induces downregulation of myogenin and reverses the course of differentiation (Mastroyiannopoulos et al., 2013). The marked increase of these transcription factors in MDC1A mouse models suggests not only that myofibroblast transdifferentiation is occurring, but that they are also playing active roles in inhibiting myogenesis.

## Contributions of Renin–Angiotensin System

Dysregulation of the RAS also contributes to the pathogenic fibrotic process via crosstalk with the TGF-beta pathway. RAS plays a systemic role across the body by regulating blood pressure, maintaining fluid and electrolyte homeostasis, and stimulating the production of aldosterone in the adrenal cortex (Sparks et al., 2014).

Renin–angiotensin system signaling splits into two pathways which act in opposition to maintain homeostasis. In the classical pathway, renin cleaves angiotensinogen to form the inactive decapeptide angiotensin I [Ang-(1–10)]. Ang I is then converted to Angiotensin II [Ang-(1–8)] by the angiotensin-converting enzyme (ACE). Ang II binds Angiotensin type 1 and type 2 receptors (AT_1_R and AT_2_R) to drive vasoconstriction, aldosterone synthesis, sodium retention, inflammation, fibrosis, and oxidative stress. Globally, the circulating levels of renin and angiotensinogen are produced by the kidney and liver, respectively (Stroth and Unger, 1999; Atlas, 2007; Sparks et al., 2014; Bavishi et al., 2016). However, individual tissues, including skeletal muscle, can generate local RAS signaling. Both global and local dysregulation of RAS signaling through Ang II leads to detrimental effects across different organ systems, contributing to cardiovascular diseases, diabetes, and kidney failure. Additionally, chronic activation of Ang II signaling is implicated in cardiac, renal, hepatic, lung, and skeletal muscle fibrosis (Kawano et al., 2000; Rodriguez-Vita et al., 2005; Cabello-Verrugio et al., 2012). Indeed, ACE, Ang II, and AT_1_R are upregulated in DMD, BMD, and MDC1A patients as well as in mouse models of these muscular dystrophies (Sun et al., 2009; Mehuron et al., 2014). Ang II upregulates *TGFB1* and *SMAD2/3* expression levels and enhances nuclear translocation of phosphorylated Smad2/3 (Fukuda et al., 2000; Rodriguez-Vita et al., 2005; Carvajal et al., 2008). Together then, these pathways converge to drive myofibroblast transdifferentiation and activation contributing to the fibrotic remodeling of the ECM.

To balance the actions of Ang II signaling, it has been demonstrated that the RAS pathway can be shunted toward formation of the heptapeptide angiotensin-(1–7) [Ang-(1–7)], which acts antagonistically to Ang II. This pathway promotes vasodilation as well as anti-inflammatory, anti-fibrotic, and anti-proliferative pathways mediated by Mas receptor signaling. Ang-(1–7) can be synthesized through three different pathways: (1) Ang II conversion to Ang-(1–7) via angiotensin-converting enzyme 2 (ACE2), (2) Ang I conversion to Ang-(1–7) through enzymes neprilysin 24.11 (NEP), thimet oligopeptidase 24.15 (TOP), or prolyl oligopeptidase 21.26 (POP), and (3) Ang I conversion by ACE2 to form angiotensin-(1–9), which can then further be converted by ACE to form Ang-(1–7). Out of these three pathways, Ang II is the major substrate for synthesis of Ang-(1–7). Therefore, the profibrotic effects of Ang II could be combatted along the ACE2 axis through increased conversion of Ang II to Ang-(1–7).

Indeed, several studies have reported beneficial anti-fibrotic effects by inducing RAS signaling toward the ACE2/Ang-(1–7)/Mas receptor axis in the skeletal muscles of dystrophic mice. In work performed by Acuna et al. (2014), inhibition of the Mas receptor in the mdx model of DMD lead to impaired muscle histopathology due to increased TGF-beta signaling and fibrosis. Infusion of Ang-(1–7) via osmotic pumps had the opposite effects: delivery of the heptapeptide improved mdx skeletal muscle morphology, including decreased inflammation and fibrosis (Acuna et al., 2014). Additionally, Ang-(1–7) treatment protected wild-type mouse skeletal muscle from the TGF-beta-induced fibrosis (Abrigo et al., 2016). Likewise, Sabharwal et al. (2014) reported similar effects in a mouse model for delta-Sarcoglycanopathy. Early intervention with oral delivery of the Ang-(1–7) peptide (TXA127) decreased oxidative stress and fibrosis in skeletal muscle of *Sgcd−/−* mice (Sabharwal et al., 2014). Collectively, these data would suggest testing Ang-(1–7) as a single-mode therapy in preclinical models of MDC1A.

## Therapeutic Strategies Targeting Fibrosis

As we have mentioned above, recent advances have elucidated that secondary pathomechanisms downstream of the primary genetic defects can become self-ruling disease drivers in their own right. As such, generating therapies targeted at arresting and/or reversing these secondary pathologies, such as fibrosis, can have a tremendous impact on the progression of the disease as well as quality of life. Despite extensive research into some of the major disease drivers, there remains no cure or treatment for MDC1A.

Most strategies that have been used to alleviate fibrosis directly or indirectly target TGF-beta signaling pathways. One such therapy that has been tested in different mouse models of MDC1A, showing remarkable amelioration of fibrosis, is Losartan. It is an FDA-approved AT_1_R blocker (ARB) that is routinely used to control hypertension in adults but is also prescribed to children. It has been shown to be a potent anti-fibrotic and anti-inflammatory agent that works in part by reducing signaling along the Ang II/AT_1_R axis, which indirectly attenuates dysregulated TGF-beta signaling. In 2012, Elbaz et al. (2012) and Meinen et al. (2012) independently published results showing Losartan treatment resulted in reduced TGF-beta signaling. In an earlier work, Cohn et al. (2007) showed in 2007 that Losartan treatment lowered the levels of thrombospondin-1 in *mdx* mice. They reasoned Losartan treatment led to prevention of thrombospondin-1-mediated activation of latent TGF-beta, likely by causing some conformational changes to the latent TGF-beta complex (Schultz-Cherry and Murphy-Ullrich, 1993). More recently, Accorsi et al. (2015) suggested that lowering of integrin-alphaV, a potent activator of TGF-beta, could be instrumental in mediating the anti-fibrotic effects of Losartan. We comprehensively showed that integrin-alphaV and its cognate beta partners were markedly downregulated in *DyW* mouse muscle treated with Losartan. More convincingly, we also showed the levels of active TGF-beta, but not its latent form, were reduced in response to Losartan treatment (Accorsi et al., 2015). These findings suggest a possible interplay of integrin-alphaV and Losartan; however, more work is warranted to establish this link. While it is less clear the relative role that thrombospondin-1 or integrin-alphaV play to activate TGF-beta in the context of fibrotic pathology in MDC1A, it is plausible that Losartan targets more than one pathway to strongly abrogate TGF-beta signaling.

While Losartan has been shown to reduce inflammation and fibrosis, it does not lead to increases in body or muscle weight in *DyW* or *Dy^2*j*^* mice (Elbaz et al., 2012; Accorsi et al., 2016). Since failure to thrive is one of the most frequent complications in MDC1A, a successful therapeutic strategy needs to improve muscle growth (Philpot et al., 1999). Therefore, it is less likely that Losartan can be a single-mode therapy for MDC1A. Pairing the anti-inflammatory/anti-fibrotic effects of Losartan with the pro-myogenic effects of IGF-1/growth hormone indeed provided a synergistic benefit as the dual therapy resulted in significant mitigation of inflammation and fibrosis that allowed for the pro-myogenic effects of IGF-1 to facilitate improved overall growth in *DyW* mice (Accorsi et al., 2016).

While we have found Losartan to be anti-myogenic in terms of terminal myotube differentiation, Ang 1–7 has the potential to actually induce muscle growth via Mas receptor-mediated mTOR activation (Morales et al., 2016), which is well known to promote protein synthesis and subsequent muscle growth. It has been shown in a model of muscle atrophy that treatment of Ang (1–7) prevents muscle mass and function loss due to disuse (Morales et al., 2016) further supporting the possibility of its role as a single-agent therapeutic to inhibit fibrosis and promote myogenesis but remains to be tested in lama2-related pathology. A pharmaceutical formulation of the Ang 1–7 peptide, TXA127, has been granted Orphan Drug designation for muscular dystrophies; however, Context Therapeutics (previously Tarix Orphan) has yet to initiate clinical trials for these indications.

Yet another compound that is known to inhibit the TGF-beta signaling pathway is Halofuginone (Juarez et al., 2017). It is a synthetic derivative of Febrifugine which is a naturally occurring alkaloid found in the roots of hydrangea plants (Zhu et al., 2009). It has been shown to decrease Smad2/3 phosphorylation and prevent fibroblast activation in the muscle tissue of *Dy^2*j*^* mice (Nevo et al., 2010). It should be noted that a phase 1B/2A trial in DMD patients was conducted with HT-100 (a chemical formulation of Halofuginone) by Akashi Therapeutics (NCT01847573). Results have not yet been released.

Interventions that are not directly targeted toward attenuation of TGF-beta signaling have also resulted in amelioration of fibrosis. Both inhibition of a BCL2 family pro-apoptotic protein BAX (Yamauchi et al., 2013) as well as inhibition of GAPDH-Siah1-mediated apoptosis with Omigapil (a deprenyl analog) (Erb et al., 2009) attenuated fibrotic pathology to some extent in preclinical models of MDC1A. Santhera Therapeutics sponsored a single-center interventional trial at the NIH to establish pharmacokinetic (PK) profile and safety/tolerability of Omigapil (NCT01805024). The trial was completed in 2018 and successfully met the primary objectives. While the PK profile of Omigapil was found suitable for further development, the next steps have not yet been announced by the company.

Genetic ablation of matricellular proteins has also been shown to ameliorate fibrosis in models of muscular dystrophy. Deletion of periostin (Lorts et al., 2012) or osteopontin (Capote et al., 2016) have been shown to be protective in mouse models of DMD. Of note, deletion of osteopontin in the severe *Dy^3*K*^* mouse model of MDC1A was not shown to be protective but rather exacerbated the pathology (Gawlik et al., 2017).

Finally, strategies such as AAV-mediated expression of mini-Agrin (Moll et al., 2001), CRISPR/CAS9-mediated over expression of laminin-alpha1 (Kemaladewi et al., 2019), or treatment with recombinant laminin-111 (Rooney et al., 2012) would be logical choices to compensate for the missing laminin-alpha2. *LAMA1* is similar to *LAMA2*, thus overexpression would compensate for lack of *LAMA2* function while minimizing the risk of an immune response to laminin-111. However, maximal translational impact might require a combination of targeting the genetic defect along with some of the secondary pathologies. This is particularly true for a disease like MDC1A because of the congenital onset of fibrosis. Therefore, it may be critical to treat fibrosis in parallel to any approach that will compensate for the missing gene. Proof of concept has been demonstrated in the context of volumetric muscle loss in mice. It has been shown that in order for regenerative therapies to be most effective, amelioration of the fibrotic signaling is required for maximal impact (Larouche et al., 2018).

## Non-Invasive Biomarkers for Fibrosis

Another critical aspect to development of therapeutics is the ability to measure changes in pathology in response to an intervention, ideally in a manner that does not involve the acquisition of muscle biopsies from patients, especially young children. This necessitates the development of non-invasisve biomarkers to measure disease. Numerous methodologies have been developed for this purpose including, but not limited to, serum biomarkers, magnetic resonance imaging (MRI), and electrical impedence myography (EIM).

In order for a biomarker to be effective, it needs to be indicative of disease process and be responsive to treatment. Serum biomarkers of fibrosis have been elucidated in models of lama2-deficient mice. Our lab showed that both latent and active levels of TGF-beta are increased in the serum of DyW mice but only levels of active TGF-beta were decreased in response to anti-fibrotic treatment (Losartan). We also showed that levels of Timp1 were overexpressed in DyW serum and were also decreased in response to Losartan (Accorsi et al., 2015).

Magnetic resonance imaging is also a robust measure of muscle mass, contractile area, inflammation, and fibrosis. We showed that pixel-by-bixel analyses of T2 MR maps were reduced in DyW mice compard to WT, indicative of fibrosis, but were rescued in response to anti-fibrotic therapy. These values correlated with changes in muscle fibrosis measured by Sirius red staining as well as collagen-1a gene expression. MR indices also validated the anti-inflammatory properties of Losartan as well as the lack of impact on muscle volume (Vohra et al., 2015).

Electrical impedance myography is another method for measuring intrinsic muscle properties and has been utilized as a pre-clinical and clinical non-invasive biomarker in numerous settings of muscle disease. Hakim et al. (2017) showed that EIM was able to measure significant difference in EIM parameters in DMD canines that significantly correlated with fibrotic build-up in the measured muscles. EIM has also been utilized in multiple clinical settings in FSHD (Rutkove et al., 2007; Mul et al., 2018), ALS (Rutkove et al., 2007, 2017), and DMD (Rutkove et al., 2017) where it was also shown to be responsive to intervention with corticosteroids. EIM was also recently measured in patients with Col6- and LAMA2-CMD showing significant changes in resistance in LAMA2-CMD patients suggestive of fibrosis (Nichols et al., 2018).

## Conclusion

*LAMA2*-related muscular dystrophy has, at its root, chronic dysregulated remodeling of the myomatrix. This remodeling results in widespread fibrosis and expression of proteins that have the ability to change the course of muscle development. It is therefore important to understand further the natural disease progression and how these processes drive the etiology of MDC1A to allow elucidation of therapeutic windows and targets. While much of this is known in preclinical models of MDC1A, there is much to be learned about the clinical course of fibrosis, which drives the devastating pathology in patients with CMDs.

## Author Contributions

AA, MC, and MG: manuscript preparation.

## Conflict of Interest

AA was employed by Fulcrum Therapeutics. MC and MG were employed by Pfizer Inc.
